# The effect of metallic substrates on the optical properties of monolayer MoSe_2_

**DOI:** 10.1038/s41598-020-61673-0

**Published:** 2020-03-18

**Authors:** M. Grzeszczyk, M. R. Molas, K. Nogajewski, M. Bartoš, A. Bogucki, C. Faugeras, P. Kossacki, A. Babiński, M. Potemski

**Affiliations:** 1 Institute of Experimental Physics, Faculty of Physics, University of Warsaw, ul. Pasteura 5, 02-093 Warsaw, Poland; 2 Laboratoire National des Champs Magnétiques Intenses, CNRS-UGA-UPS-INSA-EMFL, 25, avenue des Martyrs, 38042 Grenoble, France; 3 Central European Institute of Technology, Brno University of Technology, Purkyňova 656/123, 612 00 Brno, Czech Republic

**Keywords:** Two-dimensional materials, Two-dimensional materials, Optical spectroscopy, Two-dimensional materials, Surfaces, interfaces and thin films

## Abstract

Atomically thin materials, like semiconducting transition metal dichalcogenides (S-TMDs), are highly sensitive to the environment. This opens up an opportunity to externally control their properties by changing their surroundings. Photoluminescence and reflectance contrast techniques are employed to investigate the effect of metallic substrates on optical properties of MoSe_2_ monolayer (ML). The optical spectra of MoSe_2_ MLs deposited on Pt, Au, Mo and Zr have distinctive metal-related lineshapes. In particular, a substantial variation in the intensity ratio and the energy separation between a negative trion and a neutral exciton is observed. It is shown that using metals as substrates affects the doping of S-TMD MLs. The explanation of the effect involves the Schottky barrier formation at the interface between the MoSe_2_ ML and the metallic substrates. The alignment of energy levels at the metal/semiconductor junction allows for the transfer of charge carriers between them. We argue that a proper selection of metallic substrates can be a way to inject appropriate types of carriers into the respective bands of S-TMDs.

## Introduction

Out-of-plane quantum confinement in monolayers (MLs) of semiconducting transition metal dichalcogenides (S-TMDs), as well as their large in-plane effective masses of electrons and holes contribute to strong Coulomb interactions between charge carriers, which is reflected in large exciton binding energies^1,2^. Due to the nature of those materials, their electronic and optical properties are highly sensitive to their surroundings. This can be used as a non-invasive way to influence the screening of electron-hole Coulomb interaction in S-TMDs MLs^3–7^. On the other hand, the electronic properties of atomically thin layers can be locally altered by metals, which are contacted with the samples^8–10^. In consequence, using metals as substrates may affect the doping of S-TMD MLs due to the alignment of energy bands at the metal/semiconductor junctions. A selection of suitable substrates can be a way to inject appropriate types of carriers into the respective bands of S-TMDs. Better understanding of the role of interfaces and doping processes is important for future applications of thin S-TMD layers in a variety of modern electronic devices (field-effect transistors^11^, sensors^12^, spintronic^13^ and valleytronic circuits^14^ etc.) since all of them incorporate metallic contacts.

**Figure 1:**
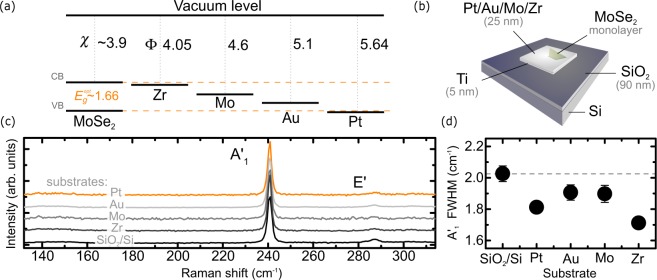
(**a**) A scheme of energy levels diagram of considered MoSe_2_/metal heterostructures. The electron affinity and metal work function are denoted with *χ* and Φ, respectively; CB and VB mark the bottom of the conduction and top of the valence bands, *E*_*g*_ is the energy band gap of MoSe_2_ ML. All values are given in electronvolts (eV). (**b**) Schematic illustration of samples under study. (**c**) Room-temperature Raman scattering spectra of monolayer MoSe_2_ on different metallic substrates, Raman spectrum of MoSe_2_ ML on Si/SiO_2_ is also added as a reference. (**d**) The comparison of A′_1_ full-width-half-maximum (FWHM) on different substrates, extracted from the fitted Lorentizan functions.

We study the effect of metallic substrate on optical properties of MoSe_2_ ML. The ground exciton state of the MoSe_2_ ML is bright^15^ and the corresponding emission spectrum comprises two peaks related to neutral and charged excitons^16,17^. Metals, on top of which the MoSe_2_ flakes were transferred, were chosen based on their fundamental physical properties: electrical and thermal conductance, work functions, and chemical stability. Platinum (Pt) and gold (Au) are often used as high-work-function electrical contacts (the work functions of Pt and Au are equal to 5.64 eV^18^ and 5.1 eV^19^, respectively). When connected to monolayer MoSe_2_ they are expected to form p-type Schottky barriers. The opposite should be observed for zirconium (Zr), characterised by low work function (equal to 4.05 eV^19^) and supposed to result in n-type Schottky contacts. We also consider molybdenum (Mo) that should form strong orbital overlaps with materials comprising the same element, in particular, MoSe_2_. A diagram representation of the energy structure of ML MoSe_2_ metal junctions under study is shown in Fig. 1(a). The investigated samples are schematically illustrated in Fig. 1(b).

## Results

Raman scattering spectra measured at room temperature on the studied structures are presented in Fig. 1(c). The Raman scattering spectrum of the MoSe_2_ ML exfoliated on Si/SiO_2_ is also shown for comparison. The spectra display two modes: an in-plane ${{\rm{E}}}^{{\prime} }$ mode at 240 cm^−1^ and an out–of–plane ${{\rm{A}}}_{1}^{{\prime} }$ mode at ~290 cm^−1^. These modes are characteristic of MoSe_2_ MLs^20^, which confirms the single-layer thickness of the investigated samples. It is well known, that the strain and disorder are great factors in shaping the properties of TMD monolayers. Their impact is well reflected in Raman scattering spectra^21–24^. As can be seen in Fig. 1(c), the Raman scattering spectra of MoSe_2_ MLs on metals and on Si/SiO_2_ are very similar. No additional features in the energy range presented, as well as no apparent broadening of the observed phonon modes (see Fig. 1(d)) suggest that the studied MLs were not significantly affected by either the metal, on which the flakes were deposited or strain and disorder that could have been introduced into the flakes during the fabrication process. Therefore both factors are not included in further analysis of our results.

**Figure 2:**
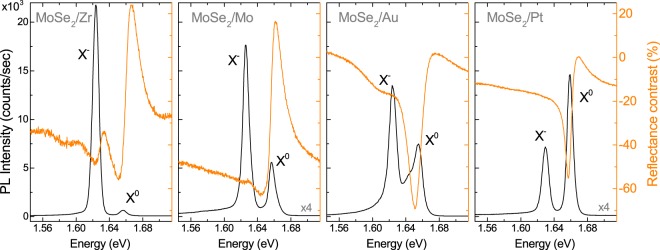
Photoluminescence (PL) and reflectance contrast (RC) spectra of ML MoSe_2_ deposited on different metallic substrates, measured at T = 5 K. Note that the vertical scales of the PL intensity and RC are set the same for all four panels.

The photoluminescence (PL) spectra, shown in Fig. 2 with black lines comprise two well-separated emission lines, which are attributed to the neutral (X^0^ ~ 1.66 eV) and the negatively charged (X^−^ ~ 1.63 eV) excitons formed in the vicinity of the so-called A exciton at the K^±^ points of the Brillouin zone^16,25^. The assignment of the trion complex to the particular charge sign (positive or negative) is not straightforward. Commercially available materials used for exfoliation, are typically unintentionally doped. Moreover, the doping can vary spatially and correlates to the presence of hydrogen in underlying substrates^26,27^. The majority of reports on MoSe_2_ states unintentional n-doping in the material^28–30^. Consequently, we adapt the same assumption. The arguments for n-type doping of the studied MLs are presented in the following section. Additionally, a third feature at around 1.65 eV can be observed in the PL spectrum of the ML deposited on the Au substrate. No similar emission peak was reported so far for MoSe_2_ MLs. A possible assignment of this peak is difficult as the contribution of phonons, dark excitons and biexcitons is not very likely. By comparing the spectra (with panels arranged by increasing metal work function from the left- to the right-hand side) an obvious trend can immediately be noticed. With increasing work function of the metal, the relative intensity of the neutral excitonic line to the charged exciton line increases. For the MoSe_2_/Zr structure, the emission-related to the neutral exciton X^0^ is approx. 40 times weaker than that of the charged exciton. On the other hand, the intensities of the X^−^ lines are about three and two times larger as compared to the X^0^ peaks for the MoSe_2_/Mo and MoSe_2_/Au structures, respectively. In the case of MoSe_2_/Pt structure, for which the metal work function is highest, the neutral exciton emission is about two times stronger as compared to the charged exciton one. An analogous effect can be recognized in the reflectance contrast (RC) results measured at T = 5 K, shown in Fig. 2 with orange lines. For three structures, *i.e*. MoSe_2_/Zr, MoSe_2_/Mo and MoSe_2_/Au, two resonances can be observed in the RC spectra, which are attributed to the charged and neutral excitons^16,17,31^. For the MoSe_2_/Pt stack, there is only one dip in the RC spectrum, which is ascribed to the neutral exciton.

**Figure 3:**
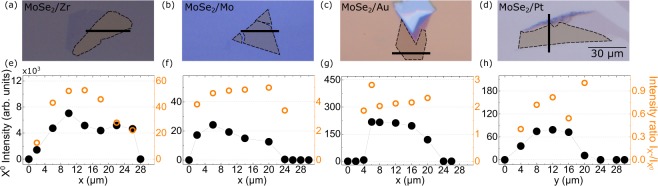
(**a**–**d**) Optical images of the investigated flakes. Dashed lines indicate the boundaries of MoSe_2_ MLs. (**e**–**h**) The neutral exciton (X^0^) intensity accompanied with the intensity ratio of the trion (X^−^) to the X^0^ line measured at T = 5 K on MoSe_2_ as a function of the position along the lines indicated in the respective images shown above.

The results described above are representative of the structures under investigation. To confirm their validity and establish homogeneity of our samples, each structure with MoSe_2_ ML of a size approximately 20 μm by 20 μm (microscopic images in Fig. 3(a–d)) was measured at several spots within the flake’s area. The intensity of the neutral exciton emission at each point along the cross-section of the MLs marked with black lines in Fig. 3(a–d) is shown in Fig. 3(e–h). The intensity ratio of the trion (X^−^) and the neutral exciton (X^0^) at the points is also shown in Fig. 3(e–h). Some differences in intensity ratios may result from defects of the substrate surface^26,32^. Moreover, a slight edge effects can be noticed, particularly in Fig. 3(e,f). The ratio decreases near the edges, which points out to the depletion of electrons in those regions. A similar effect was recently reported in MoS_2_ structures studied through the tip-enhanced Raman spectroscopy and was related to the edge states capturing electrons near the structure edges.^33^

**Figure 4:**
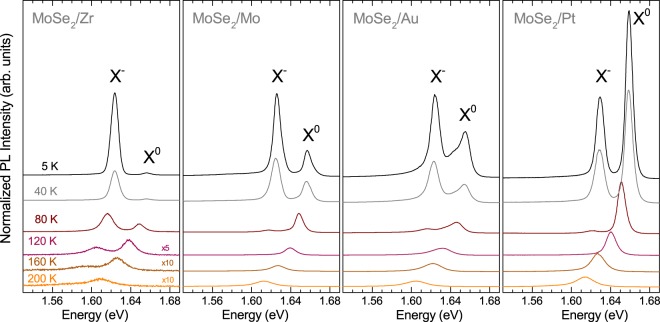
Temperature evolution of PL spectra of MoSe_2_ MLs deposited on different metallic substrates. The PL spectra are normalized to the intensity of the X^−^ line at 5 K. The spectra are vertically shifted for clarity and some of them are multiplied by scaling factors in order to avoid their intersections with the neighbouring experimental curves.

To examine the emission spectra of studied MoSe_2_ MLs in more detail, we performed PL measurements over a wide temperature range from 5 K to 300 K. It is known that increasing the temperature of typical MoSe_2_ MLs deposited on Si/SiO_2_ leads to quickly vanishing X^−^ emission^16^. Selected PL spectra are shown in Fig. 4. In order to maintain the legibility of the plot, the spectra are displaced vertically and, if needed, multiplied by a scaling factor. Two main effects of temperature can be noticed. At low temperature, the PL spectrum of the MoSe_2_/Zr sample is dominated by the trion’s contribution. The trion emission rapidly quenches as temperature increases and the emission can not be observed at T > 200 K. In the case of three other structures, *i.e*. MoSe_2_/Mo, MoSe_2_/Au and MoSe_2_/Pt, the X^−^ emission disappears from the PL spectra more quickly and it can not be recognized at T > 120 K. Finally, for all the studied structures, only the X^0^ line is apparent in the PL spectra at T > 200 K. The X^0^-exciton feature shows an overall redshift consistent with the temperature evolution of the band gap^16^.

## Discussion

The observed effects of the metallic substrates on the optical response of the MoSe_2_ ML can be explained in terms of the corresponding doping. A schematic representation of energy levels of the studied ML and the metals used as substrates is presented in Fig. 1(a). It is important to mention that being aware of a significant effect of surrounding environment of the S-TMD ML on the magnitude of its electronic band gap (*E*_*g*_)^34,35^, we decided to implement the mean value of the optical band gap (${E}_{g}^{opt}$) of the studied MLs in the former analysis of the metal/semiconductor junctions. Our approach results from the fact that the ${E}_{g}^{opt}$ value, defined as the energy difference between the *E*_*g*_ and the X^0^ binding energy (*E*_*b*_), is much less affected by the surrounding environment of the ML, as can be seen in Fig. 2. This indicates that the electronic band gap (*E*_*g*_) renormalization is almost completely compensated by the renormalization of the *E*_*b*_ resulting in a small variation of the optical band gap ${E}_{g}^{opt}$^36,37^. As can be seen in Fig. 1(a), the relative position of the Fermi levels in metals with respect to the conduction band (CB) and valence band (VB) edges in the MoSe_2_ ML changes significantly due to the variation of the metal work function. For the two metals characterised by extreme work functions, *i.e*. Zr and Pt, their Fermi levels coincide correspondingly with the extrema of the CB and VB of the MoSe_2_ ML. This may result in the creation of metal/semiconductor junctions, which exhibit n- (Zr) or p-type (Pt) characteristics and therefore permit to form, respectively, the negatively or positively charged excitons. Our observation is in good agreement with data that have recently been reported for TMD/metal interfaces^38–40^.

**Figure 5:**
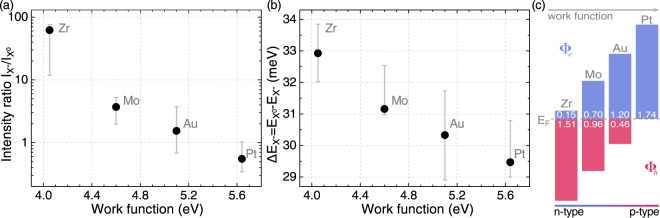
(**a**) Intensity ratio of the charged exciton to the neutral exciton line and (**b**) charged exciton binding energy ($\Delta {E}_{{X}^{-}}$) versus the metal work function. The grey error bars represent the deviation range of the values marked with solid circles (extracted from Fig. 2), obtained by analysing measurements from different spots within the flake’s area, partially shown in Fig. 3. (**c**) Comparison of the calculated Schottky Barrier Heights (Φ) for selected metal/MoSe_2_ junctions.

Let us analyse the energy of the CB and the VB extrema of MoSe_2_ ML in reference to the metals’ work functions. Similar energy values of MoSe_2_ affinity and Zr work function result in the band alignment. Electrons can easily transfer between the MoSe_2_ CB and the metal surface, shifting up the Fermi level. In that case, the structure can be characterised as a Schottky barrier, which serves as an efficient electron trap. As a consequence of that band alignment, one expects that the studied ML deposited on Zr reveals relatively high n-type doping. This leads to the appearance of the negatively charged excitons in both the PL and RC spectra (see Fig. 2). The high doping level in the MoSe_2_/Zr structure results in the observation of the X^−^ resonance in the corresponding RC spectrum measured at T = 5 K (see Fig. 2). The MoSe_2_ MLs on Mo and Au substrates are less n-type doped, but still two X^0^ and X^−^ resonances can be recognized in both corresponding RC and PL spectra (see Fig. 2). In those two cases (Mo and Au), the Fermi energy of the metal is located within the MoSe_2_ ML energy band gap. Assuming that the exfoliated MoSe_2_ crystals were intentionally undoped, their Fermi levels should be in the middle of the energy gap as in conventional semiconductors. That would amount to the energy of approx. 4.69 eV, *i.e*. close to the work functions of Mo (4.6 eV) and Au (5.1 eV). Consequently, it was expected that the MoSe_2_ MLs would remain essentially undoped when placed on Mo or Au substrates, and only the neutral exciton resonance would be observed in the RC and PL spectra. As can be seen in Fig. 2, the X^0^ and X^−^ transitions are apparent in both types of experiments, which strongly suggests that the exfoliated MoSe_2_ crystals are unintentionally n-doped. Note that the measured PL spectra of MoSe_2_ deposited on Zr, Mo, and Au substrates resemble those of typically studied MoSe_2_ samples on Si/SiO_2_ substrates^16,25,34,41^. The spectra of the MoSe_2_/Pt structure show that the neutral exciton emission is more intense than the trion one. As platinum’s work function falls within the VB of the investigated ML, the p-type doping in the MoSe_2_ ML can be expected in such a case. However, as we already discussed, the MoSe_2_ crystals used for exfoliation were probably unintentionally n-doped. The deposition of the ML on the Pt substrate results in a significant decrease of the X^−^ intensity, but does not permit to create positively charged excitons. Moreover, as it was shown in ref. ^29^, the binding energy of the negative trion is affected by electrostatically-tuned doping level to larger extent than the binding energy of the positive trion, which may also support our attribution of the lower energy feature in the PL spectra to the negative trion (see Fig. 5(b)).

 Figure 5(a,b) present the trion to neutral exciton intensity ratio (I${}_{{X}^{-}}$/I${}_{{X}^{0}}$) and the energy difference ($\Delta {E}_{{X}^{-}}={E}_{{X}^{0}}-{E}_{{X}^{-}}$) between the neutral and charged exciton emission lines. Note that $\Delta {E}_{{X}^{-}}$ can be defined as the binding (dissociation) energy of the charged exciton, which is the energy required to promote one of the trion’s electrons to the CB edge in the limit of infinitesimally small doping^42,43^. As can be seen in Fig. 5, both the intensity ratio and the trion’s binding energy systematically decrease with the increase of the metal work function. The quantitative impact of the work functions on the observed changes, shown in Fig. 5(a,b), varies considerably. While the trion binding energy changes about 10% with increasing the work function, the intensity ratio decreases more than 50 times. It is important to mention that the influence of the metallic substrate on the trion binding energy is probably accompanied by the variation of the neutral exciton binding energy ($\Delta {E}_{{X}^{0}}$), similarly as it was demonstrated for different dielectric environments of S-TMD monolayers^44,45^. However, a recent theoretical work^46^ demonstrates that the ratio of the trion to the exciton binding energy ($\Delta {E}_{{X}^{-}}$/$\Delta {E}_{{X}^{0}}$) is not fixed, but changes with the environment of the ML. In consequence, we are not able to quantitatively estimate the effect of metallic substrate on the neutral exciton binding energy.

In many practical cases, metal-semiconductor junctions can be reasonably described by a simple model relying on the Schottky Barrier Height (Φ), which is the energy, charge carriers have to overcome while being transported across the junction. The possibility of tuning Φ is highly desirable for various reasons, most of which determine the quality of electronic devices based on TMDs, especially from the viewpoint of the reduction of contact resistance^47^. The biggest difficulty in constructing efficient electrical contacts to TMD layers is so-called Fermi level pinning (FLP)^48,49^. The strength of FLP in a given semiconductor brought into contact with a set of metals of different work functions can be characterised by a slope of linear dependence fitted to the Φ-versus-*χ* data. In our case, we neglect the contribution from this effect by assuming weak interactions between the metal and the MoSe_2_ ML^39,50,51^. By using the Schottky-Mott model it is straightforward to calculate the Schottky Barrier Heights for various metal/semiconductor junctions:

$$\begin{array}{ccc}{\Phi }_{e} & = & \Phi -\chi \\ {\Phi }_{h} & = & {E}_{ip}-\Phi \end{array}$$

in which Φ_*e*_ and Φ_*h*_ are the barrier heights for electrons and holes, respectively, *χ* is the semiconductor electron affinity, and *E*_*ip*_ denotes the ionization potential. The obtained values are presented in Fig. 5(c). Our results show good agreement with the above analysis based on the relative alignment of the conduction and valence bands in the MoSe_2_ MLs and metals’ work function sketched in Fig. 1. As can be appreciated in Fig. 5, the lowest Schottky barrier height for electrons of about 0.15 eV is obtained for Zr, while the highest one, equal to about 1.74 eV, for Pt. Interestingly, the Schottky barrier height for holes is almost 0 for Pt. These simple calculations support our conclusion based on experimental results, that the type of doping in MoSe_2_ ML can be altered in a controlled way by placing it on metallic substrates with selected work functions.

## Conclusions

We have investigated the effect of metallic (Pt, Au, Mo, or Zr) substrate on the optical response of MoSe_2_ ML. It has been found that the emission intensity ratio of the charged to neutral excitons as well as the trion binding energy decrease with increasing the work function of the substrate. Our measurements reveal that the PL and RC spectra of the structure expected to exhibit the p-type characteristics (MoSe_2_/Pt) are dominated by the neutral exciton. When the Fermi level of metals falls inside the MoSe_2_ ML band gap, like for Mo and Au, both the PL and RC spectra show two resonances due to the neutral and charged excitons. On the contrary, in the structure with the metal’s work function matching the bottom of the semiconductor’s CB (MoSe_2_/Zr) strong resonances originating from the negatively charged exciton are seen in both the PL and RC spectra. We explain this effect in terms of variable doping of the MoSe_2_ ML induced by the metal substrate. The alignment of the energy levels at the metal/semiconductor junction allows for the transfer of carriers between the layers. The presented results demonstrate a doping method of ML TMDs by appropriately selecting the metallic substrates. A versatility of standard optical experimental methods like PL and RC is demonstrated. It is shown, that they can be successfully used to check the quality and characteristics of metal/semiconductor junctions.

## Methods

Metallic substrates were prepared by laser lithography and e-beam evaporation employed for patterning pieces of an Si/(90 nm)SiO_2_ wafer with a network of slabs made of 5 nm thick Ti adhesion layer followed by 25 nm thick Pt, Mo, Au, or Zr layer. MoSe_2_ MLs were prepared by all-dry PDMS-based exfoliation^52^ of bulk crystals purchased from HQ Graphene. The flakes of interest were first identified under an optical microscope and then subjected to atomic force microscopy and Raman spectroscopy characterisation to unambiguously determine their thicknesses and assess their overall quality. Right before transferring the flakes onto a chosen substrate, the substrate’s surface was cleaned with either dry CHF_3_ reactive-ion-plasma (Pt, Au, Mo) or wet HF etching (Zr) to remove the native oxide layer and other possible contaminants. A schematic representation of the samples is shown in Fig. 1(b). To verify the credibility of the obtained results, two sets of samples were produced in the same manner.

The investigated samples were placed on a cold finger of a continuous flow cryostat mounted on x-y motorized positioners. The excitation light was focused through a 50x long-working distance objective with a 0.5 numerical aperture giving a laser spot of about 1 μm diameter. The signal was collected via the same microscope objective, sent through a 0.5 m monochromator, and then detected by a CCD camera. The PL measurements were carried out using *λ* = 514.5 nm radiation from a continuous wave Ar^+^ ion laser. The excitation power focused on the sample was kept at ~50 μW during all PL measurements to avoid local heating. For the RC study, a 100 W tungsten halogen lamp was used as a source of excitation. Light from the lamp was coupled to a multimode fiber of 50 μm core diameter, and then collimated and focused on the sample to a spot of about 4 μm diameter. The RC spectra are defined as $RC(E)=\frac{R(E)-{R}_{0}(E)}{R(E)+{R}_{0}(E)}\times 100 \%$, in which *R*(*E*) and *R*_0_(*E*) is the reflectance of the sample with the MoSe_2_ ML and of the same structure without the ML, respectively. The unpolarized Raman scattering measurements were carried out in the backscattering geometry using a *λ* = 532 nm CW diode laser. The power of light on the samples did not exceed 70 μW. The collected Raman signal was dispersed by a 0.75 m spectrometer equipped with 1800 grooves/mm gratings.

## Data Availability

The datasets obtained during experiments and analysis in course of manuscript preparation are available from the corresponding author on reasonable request.
